# Citrate usage in the leading causes of blindness: new possibilities for the old metabolite

**DOI:** 10.1007/s11306-018-1377-1

**Published:** 2018-06-05

**Authors:** Marta Michalczuk, Beata Urban, Tadeusz Porowski, Anna Wasilewska, Alina Bakunowicz-Łazarczyk

**Affiliations:** 1 Department of Paediatric Ophthalmology and Strabismus, Medical University of Białystok, ul. Waszyngtona 17, 15-274 Białystok, Poland; 2 Department of Pediatrics and Nephrology, Medical University of Białystok, ul. Waszyngtona 17, 15-274 Białystok, Poland

**Keywords:** Citrate, Ophthalmology, Treatment, Biomarker

## Abstract

**Introduction:**

Citrate is an old metabolite which is best known for the role in the Krebs cycle. Citrate is widely used in many branches of medicine. In ophthalmology citrate is considered as a therapeutic agent and an useful diagnostic tool—biomarker.

**Objectives:**

To summarize the published literature on citrate usage in the leading causes of blindness and highlight the new possibilities for this old metabolite.

**Methods:**

We conducted a systematic search of the scientific literature about citrate usage in ophthalmology up to January 2018. The reference lists of identified articles were searched for providing in-depth information.

**Results:**

This systematic review included 30 articles. The role of citrate in the leading causes of blindness is presented.

**Conclusions:**

Citrate might help inhibit cataract progression, in case of questions confirm glaucoma diagnosis or improve cornea repair treatment as adjuvant agent (therapy of ulcerating cornea after alkali injury, crosslinking procedure). However, the knowledge about possible citrate usage in ophthalmology is not widely known. Promoting recent scientific knowledge about citrate usage in ophthalmology may not only benefit of medical improvement but may also limit economic costs caused by leading causes of blindness. Further studies on citrate usage in ophthalmology should continuously be the field of scientific interest.

## Introduction

Citrate is a well-known metabolite of the multidimensional role in the human organism (Iacobazzi and Infantino 2014). In mitochondria takes place citrate synthesis and then citrate becomes a substrate in Krebs cycle. The Krebs cycle provides the majority of cellular energy through ATP production. Furthermore, the energy production is also regulated by citrate. Citrate regulates the inhibition and the acceleration of enzymes significant in the processes involved in ATP production. On the other hand, citrate is involved in gluconeogenesis and lipid synthesis which absorb ATP energy.

Numerous studies have shown a wide range of citrate capabilities in human organism. Inflammation processes and insulin secretion are signaled by citrate (Infantino et al. 2011; Menga et al. 2013; Convertini et al. 2016). Citrate involvement in tumourogenesis and in the beginnings of the non-alcoholic fatty liver disease as iron recruiter is known (Catalina-Rodriguez et al. 2012; van de Wier et al. 2013). The reduction of histone acetylation and the development of neurological disorders are the result of altered transport of citrate (Wellen et al. 2009; Edvardson et al. 2013). Moreover, fluctuations in the citrate levels are considered as a useful diagnostic tool partially as a biomarker (Fraenkl et al. 2011; Michalczuk et al. 2017).

Scientists emphasise that various clinical usage of citrate might be beneficial (Iacobazzi et al. 2009; Wellen et al. 2009; Infantino et al. 2011; Fraenkl et al. 2011; Catalina-Rodriguez et al. 2012; van de Wier et al. 2013; Menga et al. 2013; Iacobazzi and Infantino 2014; Michalczuk et al. 2017). In ophthalmology, citrate is considered as a therapeutic agent and a useful diagnostic tool (Nagai et al. 2010; Fraenkl et al. 2011; Copeland et al. 2013; Zhao et al. 2015; Michalczuk et al. 2017; Baradaran-Rafii et al. 2017). Cataract and cornea treatment or glaucoma diagnosis might be improved by citrate usage.

### The role of citrate in the cataract inhibition

Cataract is the second cause of visual impairment and the leading course of blindness in the world (Pascolini and Mariotti 2012). According to WHO 33% of visual impairments are caused by the cataract. Visual impairments in cataract are the result of the reduced transparency of the optical medium—the lens (Truscott and Friedrich 2016). Cataract is associated with older age, men, lower household income, lower education, hypertension and diabetes (Park et al. 2016). In order to treat the cataract effectively, patients can undergo surgery (Pescosolido et al. 2016). Therefore, the possibility to inhibit cataract progression by the use of non-surgical treatment is the subject of intensive investigations (Nagai et al. 2010; Goulet et al. 2011).

**Fig. 1:**
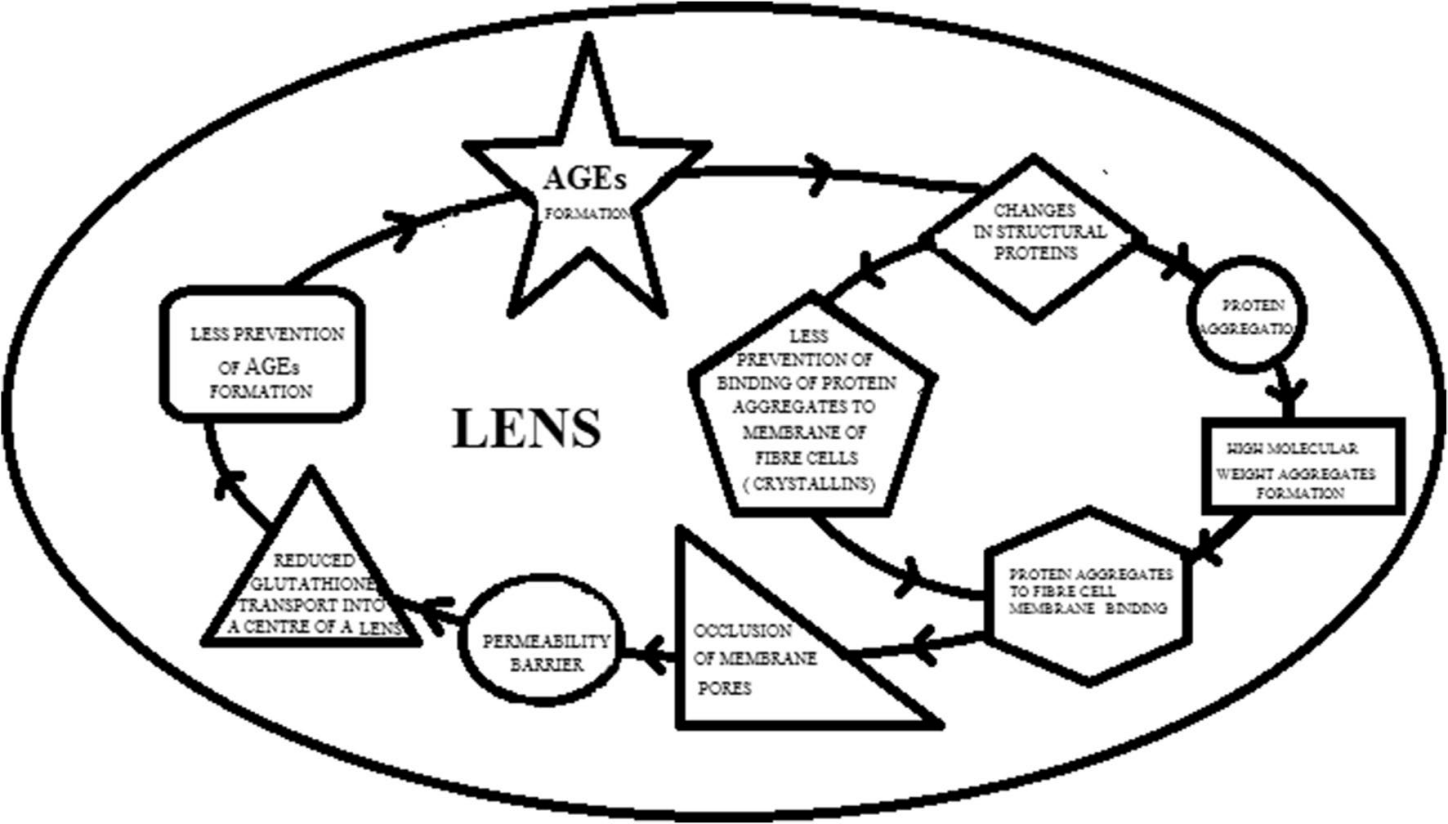
The presentation of AGEs formation mechanism and its role in cataract progression. The emergence of AGEs is the result of ageing. AGEs induce irreversible changes in structural proteins of the lens. Changes in structural proteins of the lens lead to protein aggregation and formation of high molecular weight aggregates. Moreover, also the role of structural proteins—crystallins as protective agents is changed (altered prevention of protein aggregates to fibre cell membranes binding). Protein aggregates are binded to fibre cell membranes. The membrane binding of protein aggregates cause the occlusion of membrane pores and afterwards the creation of a permeability barrier. The permeability barrier prevents a normal rate of glutathione transport into the centre of the lens. Glutathione as the antioxidant prevents formation of AGEs. Thereby, the altered glutathione transport is resulting in further AGEs formation

The development of cataract might be possibly inhibited by the use of citrate. Citrate not only inhibits formation of advanced glycation end products (AGEs) (2 g/L), but also unfolding and aggregation of the crystallins (250 mM). Therby, citrate influences on the multifactorial pathophysiology of the cataract (Fig. 1). AGEs induce irreversible changes in structural proteins of the lens (Nagai et al. 2010; Truscott and Friedrich 2016). Proteins in the lens are present for life (Lynnerup et al. 2008). They do not turn over. They degrade. Changes in structural proteins of the lens lead to protein aggregation and formation of high molecular weight aggregates (Goulet et al. 2011; Truscott and Friedrich 2016; Pescosolido et al. 2016). Protein aggregates are binded to fibre cell membranes. The membrane binding of protein aggregates may cause the occlusion of membrane pores and afterwards the creation of a permeability barrier. The permeability barrier might prevent a normal rate of glutathione transport into the centre of the lens. Glutathione is the main cellular antioxidant. Glutathione as the antioxidant (a nucleophilic scavenger) prevents formation of AGEs. Moreover, structural proteins—crystallins have not only AGEs induced the surface charge alter but also the role of crystallins as protective agents is changed. Crystallins are not only structural protein but also chaperones—they prevent binding of protein aggregates to fibre cell membranes. Therefore, the inhibitions of unfolding and aggregation of the crystallins and AGEs formation by citrate leads to conclusion that citrate implicitly may influence on many factors responsible for cataract (Nagai et al. 2010; Goulet et al. 2011). Therefore, the role of citrate usage as an effective therapy in cataract progression might be a promising possibility.

### The role of citrate in the corneal repair process

Corneal diseases have become the second greatest cause of blindness in the world (Zhao et al. 2015). Recent studies have shown that corneal repair process may benefit from citrate usage (Copeland et al. 2013; Zhao et al. 2015; Baradaran-Rafii et al. 2017). The positive effects of topical usage of citrate was proven in the therapy of several corneal diseases—a therapy of ulcerating cornea after alkali injury or in crosslinking procedure as an adjuvant agent. The therapeutic aim of citrate might be the stromal breakdown prophylaxis (Pfister et al. 1988; Copeland et al. 2013; Baradaran-Rafii et al. 2017).

**Fig. 2:**
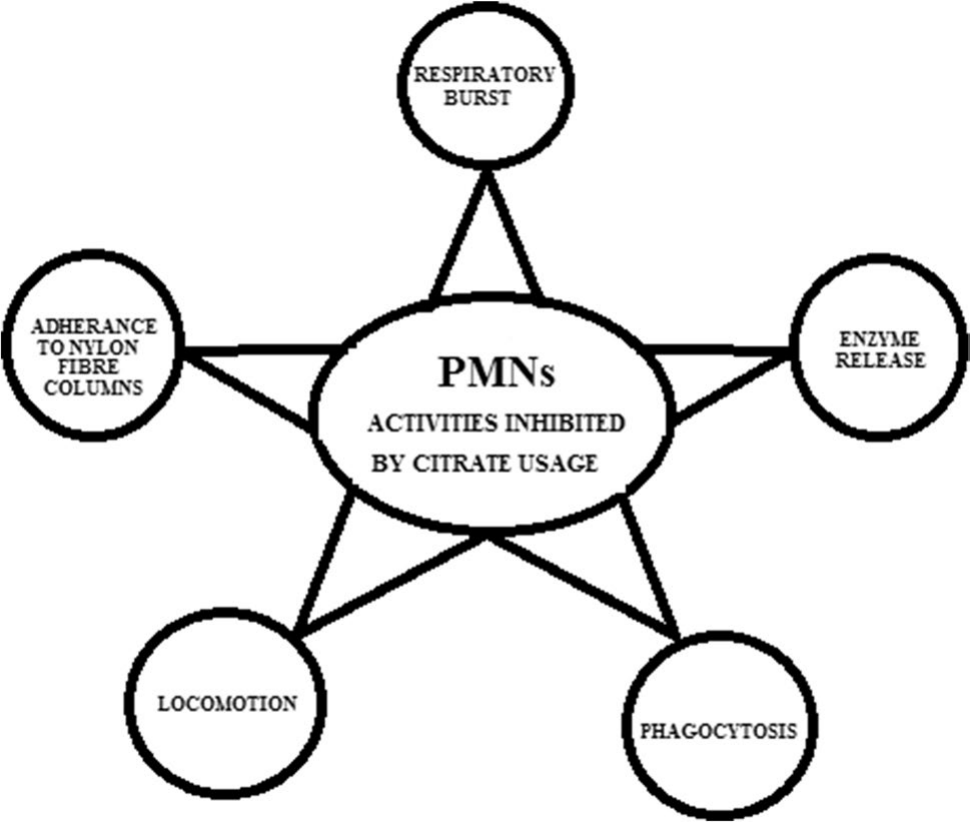
The presentation of polymorphonuclear leukocytes (PMNs) activities inhibited by citrate usage. Citrate inhibits the respiratory burst, the enzyme release, the phagocytosis, the locomotion of PMNs and the adherence of PMNs to nylon fiber columns

The positive effect of citrate appears to be related to its ability to inhibit polymorphonuclear leukocytes (PMNs) activities (Fig. 2) (Parker et al. 1985; Pfister et al. 1988; Baradaran-Rafii et al. 2017). PMNs are the predominant cell type observed in the ulcerating cornea after alkali injury. PMNs are also the only cell type present up to 2 weeks after the alkali injury. Citrate inhibits the respiratory burst, enzyme release, phagocytosis and locomotion of PMNs. Afterwards the adherence of PMNs to nylon fiber columns is prevented by citrates. That might suggest that in the blood vessels of the conjunctiva and in limbal arcades the adherence of PMNs to vascular endothelium might also be inhibited by citrates. Each of PMNs activities can be activated by different mediators. Those mediators vary in their sensitivity to divalent cations and citrate is acting as a chelator of those cations. Citrate (15 mM) exhibits great chelation activity, reducing the “free” calcium concentration from 1.3 × 10^3^ M to 2.5 × 10^5^ M. At concentrations that chelate calcium citrate also inhibits the increased oxygen consumption and the release of myeloperoxidase by PMNs stimulated by opsonized zymosan. The effect of citrate usage in treatment of corneal ulcer after alkali injury is highly promising—numbers of perforations is reduced and stability prior to perforation is increased.

The positive effect of citrate usage has been observed also during performing crosslinking procedure (Zhao et al. 2015). The crosslinked collagen–citrate films had better biomechanical properties than non-modified films. The complete epithelialization and the transparency were restored quickly. The suture during operation by the means of collagen–citrate films was tolerated. A good ability of epithelial and stromal repair were achieved. No inflammation and corneal neovascularization were observed at 6 months. Therefore, crosslinked collagen–citrate films might be considered as a highly promising biomaterials.

### The role of citrate in the glaucoma diagnosis

**Fig. 3:**
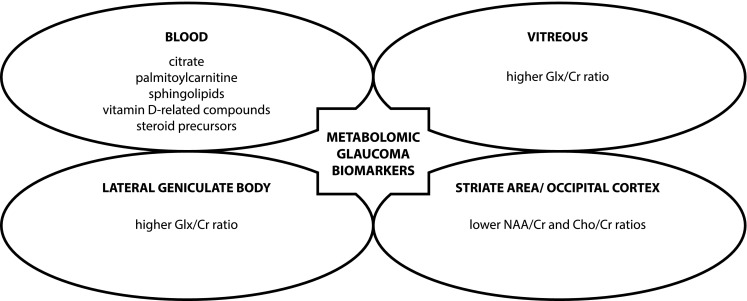
The presentation of the possible metabolomic glaucoma biomarkers. The metabolic pathways that involve citrate, palmitoylcarnitine, sphingolipids, vitamin D-related compounds, and steroid precursors were found in blood plasma. The high glutamine–glutamate (Glx)/creatine (Cr) ratio was observed in the vitreous and lateral geniculate body. The lower *N*-acetylaspartate (NAA)/Cr and choline (Cho)/Cr ratio was found in the occipital cortex and striate areas

Glaucoma is a group of chronic, progressive optic neuropathies which are marked with atrophy of the optic nerve and destruction of retinal ganglion cells (RGCs) and their axons (The American Academy of Ophthalmology 2015). The process of the optic nerve atrophy and ganglion cells damage lead to irreversible changes in visual field and even to blindness. Proper screening procedures giving early recognition of glaucoma are sufficient for counteracting the progression of the disease and the effective treatment. Nowadays, according to the American Academy of Ophthalmology (AAO) proper glaucoma screening should consist mainly of the intraocular pressure measurements, the visual field testing, assessing the optic nerve head and retinal nerve fiber layer (Fig. 3). However, identifying a reliable indicator for glaucoma appears to be in great demand (Golubnitschaja et al. 2010; Fraenkl et al. 2011; Kokotas et al. 2012; Michalczuk et al. 2017; Barbosa-Breda et al. 2018).

Citrate is one type of organic molecules considered as glaucoma biomarker (Fraenkl et al. 2011; Michalczuk et al. 2017). Studies in adult and children population showed low plasma citrate level in patients with glaucoma diagnosis (adults 104.8–23.2 vs. 128.2–31.1 mmol/L, children 16.33 ± 4.51 mg/L vs. 19.11 ± 3.66 mg/L). The role of citrate as a glaucoma biomarker results from impaired mitochondrial function contributing to glaucoma pathogenesis (Michalczuk et al. 2017). Citrate is synthesized in mitochondria from acetyl-CoA and oxaloacetate by citrate synthase and then citrate becomes a substrate in the tricarboxylic acid (TCA) cycle. The TCA oxidation process provides the major source of cellular adenosine triphosphate (ATP) production. Moreover, citrate is also a key regulator of energy production while it inhibits and accelerate enzymes significant in the processes of glycolysis, Krebs cycle, gluconeogenesis, and fatty acids synthesis. ATP is necessary for proper functioning of nerves, including the optic nerve. ATP deficiency and oxidative stress contribute to dysfunction of mitochondria in RGCs and lead to RGCs apoptosis which is regarded to be the pathological feature of glaucoma. In the light of the fact that citrate level is dependent on mitochondrial function the role of citrates as a useful diagnostic tool in glaucoma seems to be a relevant issue (Fraenkl et al. 2011; Michalczuk et al. 2017).

## Overall conclusions

The population growth and the increasing longevity will result in a greater number of elderly people with visual loss and blindness in the immediate future (Foster 2000). From 40 to 45 million persons who are blind many will need a social support. Fortunately, 80% of global blindness is avoidable. Until 2020 the international community will probably double costs allocated to the prevention of blindness. Therefore, the improvement of diagnostic procedures and treatment methods of the leading causes of blindness like cataract, glaucoma or corneal diseases seems to be the relevant issue. Taking into account overcoming the leading causes of blindness, citrate usage might be beneficial as a promising adjuvant therapeutic agent or diagnostic tool (Nagai et al. 2010; Fraenkl et al. 2011; Copeland et al. 2013; Zhao et al. 2015; Michalczuk et al. 2017; Baradaran-Rafii et al. 2017). However, citrate usage will not replace cataract surgery, keratoplasty, crosslinking or diagnostic methods for detecting glaucoma.

Scientists were looking at citrate for a lot of different medical uses (Wellen et al. 2009; Nagai et al. 2010; Fraenkl et al. 2011; Infantino et al. 2011; Catalina-Rodriguez et al. 2012; van de Wier et al. 2013; Copeland et al. 2013; Edvardson et al. 2013; Menga et al. 2013; Iacobazzi and Infantino 2014; Zhao et al. 2015; Michalczuk et al. 2017; Baradaran-Rafii et al. 2017). However, the literature about citrate usage in ophthalmology is limited (Nagai et al. 2010; Fraenkl et al. 2011; Copeland et al. 2013; Singh et al. 2013; Zhao et al. 2015; Michalczuk et al. 2017; Baradaran-Rafii et al. 2017). Diet enrichment with citrate, topical usage of citrate or plasma citrate level measurement have been already presented as beneficial in ophthalmology. Nagai et al. suggested that intake of citrate from citrus fruits can inhibit the progression of cataract as diabetes complication (2010). In the study, oral administration of citrate to diabetic rats inhibited accumulation of AGEs in lens protein. The development of cataract was delayed. Singh et al. highlighted the role of topical citrate in the management in ocular chemical injuries (2013). Zhao et al. (2015) emphasized that citrate might be helpful as a topical adjuvant during crosslinking treatment. However, no reports of oral or topical citrate usage in humans have been published to date (Nagai et al. 2010; Copeland et al. 2013; Zhao et al. 2015; Baradaran-Rafii et al. 2017). The role for citrate as adjunctive treatments in the ulcerating cornea after alkali injury, a promising adjuvant agent in crosslinking therapy or cataract inhibition agent have been proven only in animal studies. The role of the plasma citrate level as glaucoma biomarker was also described (Fraenkl et al. 2011; Michalczuk et al. 2017). Low citrate plasma level may ensure of glaucoma detection with a sensitivity of 66.7% and a specificity of 71.4% (Fraenkl et al. 2011). In comparison, a sensitivity of PSA which is widely used in prostate cancer detection is equal only 20.5% (Ankerst and Thompson 2006). Moreover, plasma citrate level is a promising glaucoma biomarker in both adult and children age groups (Fraenkl et al. 2011; Michalczuk et al. 2017).

Despite encouraging results achieved in studies on citrate usage in ophthalmic diseases, knowledge about possible citrate usage in ophthalmology is not widely known (Nagai et al. 2010; Fraenkl et al. 2011; Copeland et al. 2013; Singh et al. 2013; Zhao et al. 2015; Michalczuk et al. 2017; Baradaran-Rafii et al. 2017). The amount of research papers touching on the theme of effectiveness of treatment or diagnosis of ophthalmic diseases by the means of citrate is continuously small. Despite a huge potential and the widespread use of citrate in many branches of medicine, possibility of potential citrate usage in ophthalmology is still undervalueted (Wellen et al. 2009; Nagai et al. 2010; Fraenkl et al. 2011; Infantino et al. 2011; Catalina-Rodriguez et al. 2012; van de Wier et al. 2013; Copeland et al. 2013; Edvardson et al. 2013; Menga et al. 2013; Iacobazzi and Infantino 2014; Zhao et al. 2015; Michalczuk et al. 2017; Baradaran-Rafii et al. 2017). Although, available research papers show that citrate might help inhibit cataract progression only by changing dietary habits, in case of questions confirm glaucoma diagnosis or improve cornea repair treatment (Nagai et al. 2010; Fraenkl et al. 2011; Copeland et al. 2013; Singh et al. 2013; Zhao et al. 2015; Michalczuk et al. 2017; Baradaran-Rafii et al. 2017). Profits achieved by the means of citrate usage might not only concern about medical improvement. Early recognition and more effective treatment of leading causes of blindness might limit a number of people with visual disability and thereby limit economic costs in the branch of ophthalmology (Foster 2000). At the time when international community spends 80 million USD per year on blindness prevention it seems significant to spread recent scientific knowledge about citrate usage in ophthalmology. Hopefully, the role of citrate in ophthalmology as a therapeutic agent and a useful diagnostic tool will be the field of increasing interest, and a fortiori, further studies on citrate capability in various parts of the eye will be performed.
